# The impact of extracorporeal membrane oxygenation on the exposure to isavuconazole: a plea for thorough pharmacokinetic evaluation

**DOI:** 10.1186/s13054-022-04093-y

**Published:** 2022-07-27

**Authors:** Beatrijs Mertens, Joost Wauters, Yves Debaveye, Niels Van Regenmortel, Karlien Degezelle, Philippe Meersseman, Greet Hermans, Christophe Vandenbriele, Ruth Van Daele, Isabel Spriet

**Affiliations:** 1 Department of Pharmaceutical and Pharmacological Sciences, KU Leuven, Leuven, Belgium; 2 Pharmacy Department, University Hospitals Leuven, Leuven, Belgium; 3 Department of Microbiology, Immunology and Transplantation, KU Leuven, Leuven, Belgium; 4 Medical Intensive Care Unit, University Hospitals Leuven, Leuven, Belgium; 5 Department of Cellular and Molecular Medicine, KU Leuven, Leuven, Belgium; 6 Intensive Care Unit, University Hospitals Leuven, Leuven, Belgium; 7 Department of Intensive Care Medicine, Ziekenhuis Netwerk Antwerpen, ZNA Stuivenberg, Antwerp, Belgium; 8 Department of Perfusion Technology, University Hospitals Leuven, Leuven, Belgium; 9 Department of General Internal Medicine, Medical Intensive Care Unit, University Hospitals Leuven, Leuven, Belgium; 10 Department of Cardiovascular Diseases, University Hospitals Leuven, Leuven, Belgium; 11 Department of Adult Intensive Care, Royal Brompton & Harefield NHS Foundation Trust, London, UK

**Keywords:** Extracorporeal membrane oxygenation, Azoles, Isavuconazole, Pharmacokinetics, Therapeutic drug monitoring

Extracorporeal membrane oxygenation (ECMO) is increasingly used to provide temporary (cardio)pulmonary support in patients with life-threatening respiratory and/or cardiac failure, including critically ill patients with influenza- and coronavirus disease 2019 (COVID-19)-associated acute respiratory distress syndrome. Critically ill patients often exhibit altered and variable pharmacokinetics (PK) of antimicrobials owing to pathophysiological alterations (e.g., fluid shifts, hypoalbuminemia, renal dysfunction and augmented renal clearance) and extracorporeal treatments. ECMO might significantly affect the PK of drugs due to hemodilution from circuit priming and drug sequestration in the ECMO circuit. The impact of ECMO on the PK of mold-active triazoles, such as voriconazole and isavuconazole, has become increasingly important as they are recommended as (first-line) antifungal therapies for influenza- and COVID-19-associated pulmonary aspergillosis. Based on the high lipophilicity and extensive plasma protein binding of isavuconazole, the triazole is theoretically prone to adsorption to ECMO circuits and subsequent reduction in plasma concentrations. To date, isavuconazole exposure in ECMO patients has only been documented in two case reports and a case series (*n* = 3) in which reduced plasma concentrations during ECMO have been suggested [1–3]. In this correspondence, we would like to emphasize that the suggestion of reduced isavuconazole exposure due to ECMO *as such* should be interpreted cautiously and that additional studies are needed to evaluate the independent impact of ECMO on the PK of isavuconazole. This is in accordance with the mold-active triazoles voriconazole and posaconazole, for which drug sequestration into the ECMO circuit has been suggested by ex vivo studies and case reports. However, an independent effect of ECMO could not be confirmed in larger retrospective [4] or prospective studies [5].

We here report isavuconazole trough concentrations (*C*_min_), which were measured during routine care in four critically ill patients with concomitant isavuconazole and veno-venous ECMO treatment (approval from the local Ethics Committee; S65215). For each patient, information on ECMO and isavuconazole treatment is depicted in Fig. 1. Demographic and clinical characteristics are summarized in Additional file 1.

**Fig. 1:**
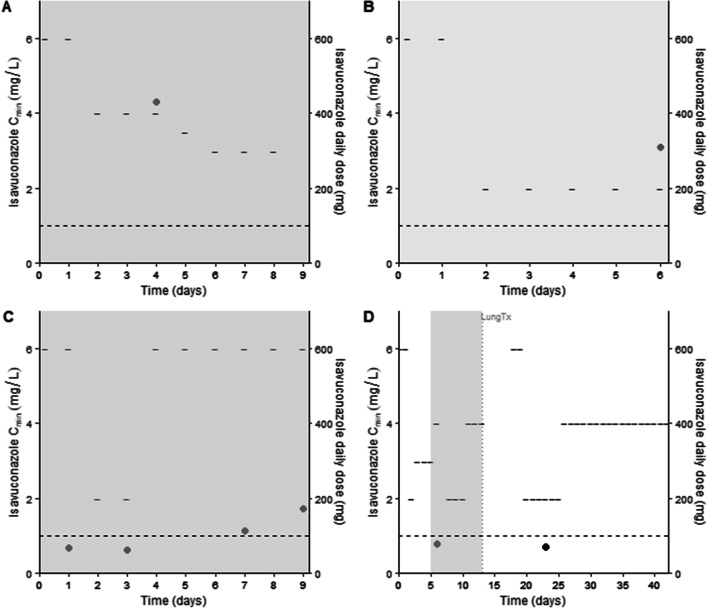
Treatment course of cases **A**, **B**, **C** and **D**. Grey shaded area: extracorporeal membrane oxygenation support; points: isavuconazole trough concentrations (mg/L); black short lines: isavuconazole daily doses (mg); black dashed horizontal line: minimal isavuconazole trough concentration threshold of 1 mg/L, based on the European Committee on Antimicrobial Susceptibility Testing breakpoints for *Aspergillus fumigatus, A. flavus* and *A. terreus*; grey dashed vertical line: lung transplantation. *C*_min_: trough concentration; *T*_x_: transplantation

In our case series, isavuconazole exposure was highly variable and four *C*_min_ were lower than 1 mg/L, which can be advocated as the minimum *C*_min_ threshold, based on the European Committee on Antimicrobial Susceptibility Testing breakpoints for *Aspergillus fumigatus, A. flavus* and *A. terreus*. Multiple factors might contribute to the variability in isavuconazole *C*_min_, including administered doses, treatment duration and time needed to reach steady state after therapy initiation/dose adjustment. Dose-corrected *C*_min_ are presented in Additional file 1: file 2. The *C*_min_ in cases A and B suggest that adequate isavuconazole exposure can be achieved during ECMO support with a standard dosing regimen. In case A, a *C*_min_ of 4.3 mg/L was reached with an increased maintenance dose of 200 mg q12h. Considering the linear PK of isavuconazole, it could be hypothesized that a standard dose of 200 mg q24h would have resulted in a *C*_min_ > 1 mg/L. In case B, this minimal target *C*_min_ was achieved with a standard maintenance dose. In contrast, the isavuconazole *C*_min_ in cases C and D did not exceed the target of 1 mg/L when correcting for the standard maintenance dose of 200 mg q24h. The latter results are in line with the previous reports by Zhao et al. [3] and Miller et al. [1], in which subtherapeutic exposure following a standard dosing regimen was documented and ascribed to ECMO *as such*.

Unfortunately, based on the previously published reports [1–3] and our case series, the independent impact of ECMO on isavuconazole exposure in critically ill patients cannot be assessed. Therefore, the key question whether subtherapeutic isavuconazole exposure in ECMO patients is caused by ECMO or by critical illness itself remains unanswered. This evidence gap underlines the need for large PK evaluations in critically ill patients, including those with augmented renal clearance, hypoalbuminemia, hepatic and renal dysfunction, renal replacement therapy and ECMO. Pending additional data (*e.g.,* ICONIC study, ClinicalTrials.gov: NCT04777058), therapeutic drug monitoring of isavuconazole is warranted in critically ill patients, both in ECMO and non-ECMO patients.

## Supplementary Information

**Additional file 1.** **file 1**: Baseline characteristics of patients included in the retrospective analysis of isavuconazole trough concentrations during extracorporeal membrane oxygenation (n= 4). **file 2**: Ratio of isavuconazole trough concentrations to isavuconazole daily doses for patients concomitantly treated with isavuconazole and extracorporeal membrane oxygenation (n= 4).

## Data Availability

Individual participant data that underlie the results reported in this manuscript are available from the corresponding author upon reasonable request, providing the request meets the local ethical and research governance criteria after publication. Patient data will be anonymized and study documents will be redacted to protect the privacy of participants.

## Abbreviations

*C*_min_: Trough concentration
ECMO: Extracorporeal membrane oxygenation
PK: Pharmacokinetic
*T*_x_: Transplantation
