# Supplementary material for: The impact of extracorporeal membrane oxygenation on the exposure to isavuconazole: a plea for thorough pharmacokinetic evaluation
Source: Crit Care. 2022 Jul 27;26:227. doi: 10.1186/s13054-022-04093-y (PMC9325953; doi:10.1186/s13054-022-04093-y)
Supplement: Supplementary file 1 — Additional file 1. file 1: Baseline characteristics of patients included in the retrospective analysis of isavuconazole trough concentrations during extracorporeal membrane oxygenation (n= 4). file 2: Ratio of isavuconazole trough concentrations to isavuconazole daily doses for patients concomitantly treated with isavuconazole and extracorporeal membrane oxygenation (n= 4). [file 13054_2022_4093_MOESM1_ESM.pdf]

Additional file 1. Baseline characteristics of patients included in the retrospective analysis of isavuconazole trough concentrations during extracorporeal membrane oxygenation (*n*= 4).

	Case A	Case B	Case C	Case D
Demographics				
Age, *years*	61	59	65	38
Sex	Male	Male	Male	Male
BMI, *kg/m²*	26,7	20,0	29,2	24,7
Clinical characteristics				
Length of ICU stay, *days*	35	35	170	63
SOFA score(s) on day(s) of isavuconazole C_{min} measurement	19	11	19 – 18 – 16 – 16	10 – 4
CRRT during isavuconazole therapy	Yes	No	Yes	No
Total duration of CRRT*, days*	27	NA	43	NA
Indication of ICU admission	COVID-19 pneumonia	Complications after lung Tx	COVID-19 pneumonia	COVID-19 pneumonia
Deceased during ICU admission	Yes	Yes	Yes	No
ECMO				
Duration of ECMO before initiation of isavuconazole, *days*	16	6	24	NA
Duration of ECMO support, *days*	27	14	67	9
ECMO circuit changes[a]	No changes	Day 8*	Days 31, 49, 57, 64*	No changes
Isavuconazole				
Indication	Probable CAPA[b]	Probable IPA	Proven CAPA[b]	Probable CAPA[b]
Therapy duration, *days*	9	7	10	43[c]

BMI: Body Mass Index; CAPA: COVID-19-associated pulmonary aspergillosis; COVID-19: coronavirus disease 2019; CRRT: continuous renal replacement therapy; ECMO: extracorporeal membrane oxygenation; ICU: intensive care unit; IPA: invasive pulmonary aspergillosis; *n*: number of patients; NA: not applicable; SOFA: Sequential Organ Failure Assessment; Tx: transplantation

[a] The days of ECMO circuit changes are calculated in relation to the initiation of ECMO support. The asterisks indicate changes of the oxygenator of the ECMO circuit.

[b] The classification was performed according to the 2020 European Confederation of Medical Mycology (ECMM) and the International Society of Human and Animal Mycology (ISHAM) consensus criteria for COVID-19-associated pulmonary aspergillosis [1].
[c] The isavuconazole therapy was interrupted for four days.

Additional file 2. Ratio of isavuconazole trough concentrations to isavuconazole daily doses for patients concomitantly treated with isavuconazole and extracorporeal membrane oxygenation (*n*= 4).

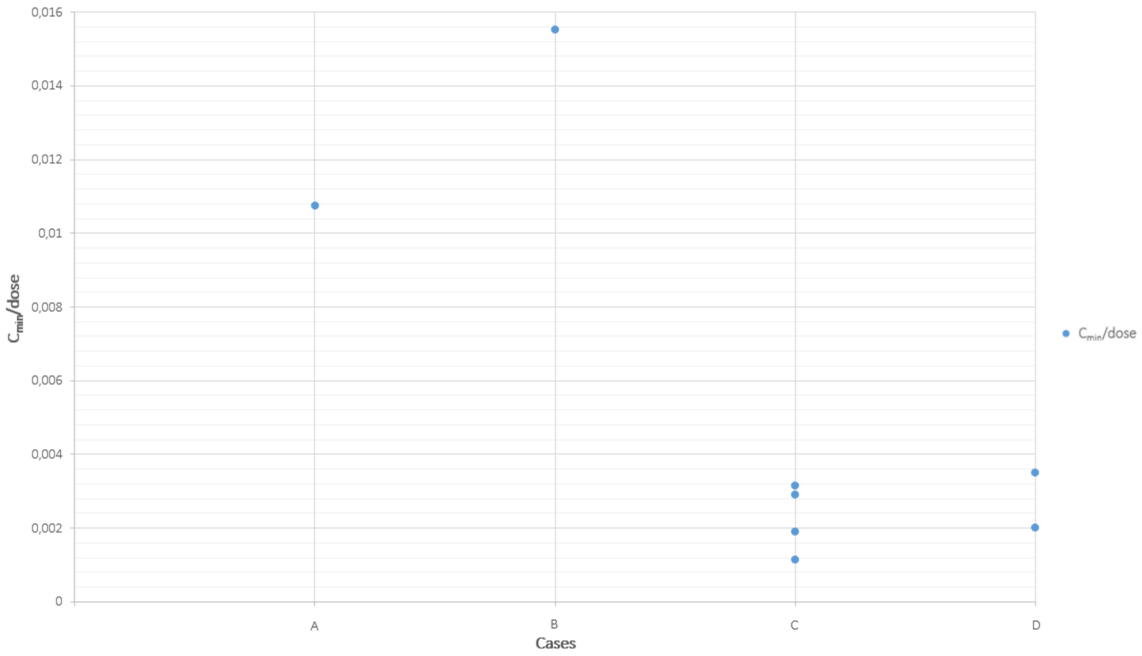

Blue point: ratio of the isavuconazole trough concentration (C_{min}) (mg/L) to the isavuconazole daily dose (mg), administered in the 24-hour period prior to blood sampling.

References

1. Koehler P, Bassetti M, Chakrabarti A, Chen SCA, Colombo AL, Hoenigl M, Klimko N, Lass-Flörl C, Oladele RO, Vinh DC *et al*: **Defining and managing COVID-19-associated pulmonary aspergillosis: the 2020 ECMM/ISHAM consensus criteria for research and clinical guidance**. *Lancet Infect Dis* 2021, **21**(6):e149-e162.
